# Diffuse Pulmonary Meningotheliomatosis Presenting as Innumerable Pulmonary Micronodules: A Case Report

**DOI:** 10.1111/1759-7714.70172

**Published:** 2025-10-07

**Authors:** Eitetsu Koh, Yasuo Sekine, Hodaka Oeda, Tadao Nakazawa

**Affiliations:** ^1^ Department of Thoracic Surgery Tokyo Women's Medical University Yachiyo Medical Center Yachiyo Chiba Japan; ^2^ Department of Pathology Tokyo Women's Medical University Yachiyo Medical Center Yachiyo Chiba Japan

## Abstract

Diffuse pulmonary meningotheliomatosis (DPM) is a rare lung condition characterized by widespread meningothelial‐like nodules and may radiologically mimic metastatic or granulomatous disease. We report a 2019‐onset case of a 44‐year‐old woman with incidentally detected, bilateral 1–2‐mm pulmonary micronodules on screening CT. Laboratory tests and pulmonary function were normal. Owing to the minute, peripheral distribution of the nodules, bronchoscopic biopsy was not feasible, and diagnostic video‐assisted thoracoscopic wedge resection was undertaken. Histology showed spindle‐to‐ovoid cells in whorled arrangements without atypia or mitoses. Immunohistochemistry revealed diffuse vimentin positivity and negativity for epithelial and neuroendocrine markers (EMA, cytokeratin AE1/AE3, SMA, chromogranin). Postoperative brain MRI showed no intracranial lesion. The patient has remained asymptomatic without radiologic progression over 3 years. This case underscores DPM as an uncommon yet important differential diagnosis of diffuse pulmonary micronodules and highlights the need for histopathologic confirmation when bronchoscopic sampling is impracticable. Where available, PR and SSTR2A immunostains may further support the diagnosis.

## Graphical abstract

Diffuse pulmonary meningotheliomatosis (DPM) presenting as innumerable 1–2‐mm bilateral micronodules in an asymptomatic 44‐year‐old woman. Bronchoscopic biopsy was infeasible; VATS wedge resection confirmed meningothelial‐like nodules (vimentin+, EMA/AE1‐AE3/chromogranin/SMA−). Brain MRI showed no intracranial lesion. Three‐year surveillance: stable disease.

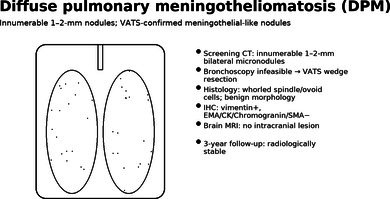

## 1 Introduction

Diffuse pulmonary meningotheliomatosis (DPM) represents the diffuse form of minute pulmonary meningothelial‐like nodules and is typically detected incidentally in middle‐aged women [1, 2]. Radiologically, DPM manifests as innumerable, bilateral micronodules that can mimic hematogenous metastases or granulomatous disease, creating a diagnostic pitfall [1, 2]. By contrast, primary pulmonary meningioma (PPM) usually presents as a solitary, well‐circumscribed nodule [3, 4]. Histologically, meningothelial‐like nodules show whorled spindle‐to‐ovoid cells; the usual immunophenotype includes positivity for vimentin, EMA, PR, and SSTR2A with negativity for cytokeratins and neuroendocrine markers [5, 6]. We report a case consistent with DPM and discuss the differential diagnosis, immunohistochemical workup, and practical considerations for follow‐up.

## 2 Case Report

**FIGURE 1:**
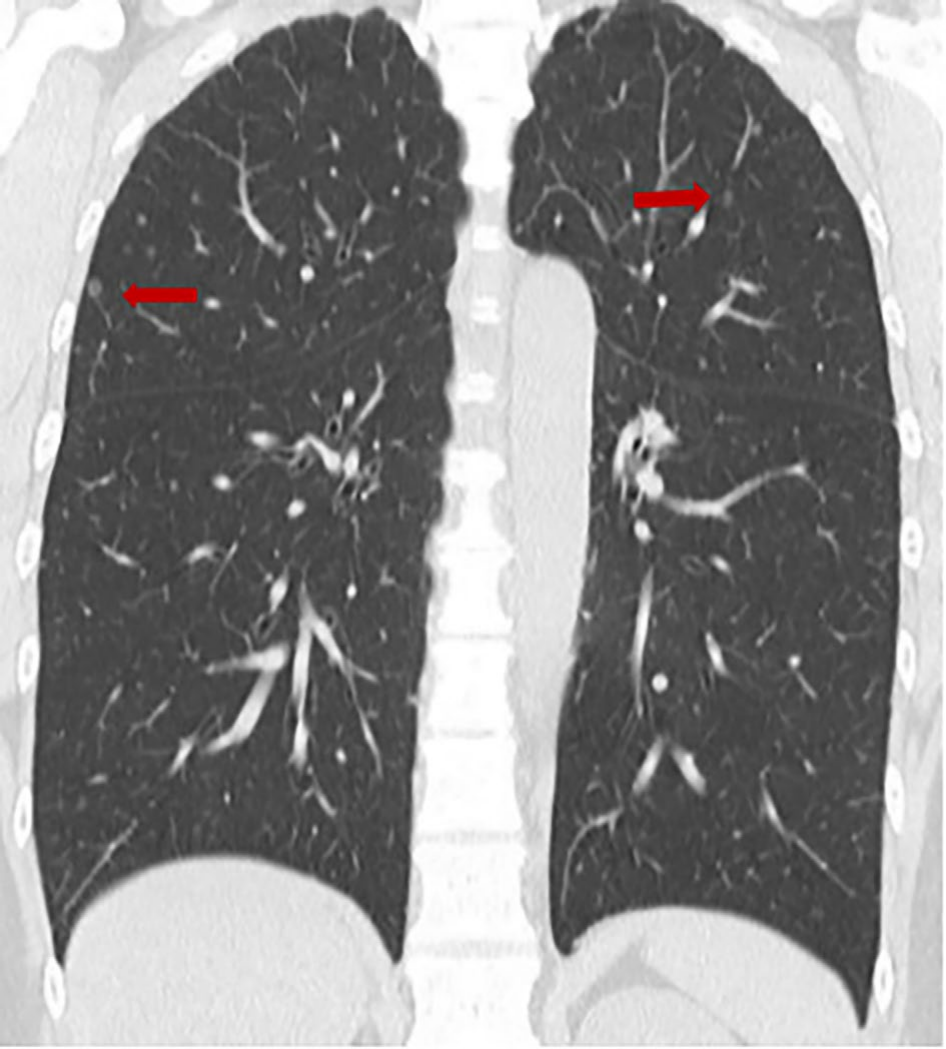
Chest CT demonstrating innumerable 1–2‐mm micronodules diffusely distributed in both lungs, predominantly peripheral/subpleural (arrows).

A 44‐year‐old woman was referred in 2019 after routine chest CT revealed innumerable, well‐defined 1–2‐mm nodules diffusely distributed in both lungs, predominantly peripheral/subpleural (Figure 1A–C). She was asymptomatic; physical examination, complete blood count, biochemistry, and tumor markers (CEA 0.9 ng/mL, SCC 1.2 ng/mL, CYFRA 0.9 ng/mL, proGRP 31.2 pg/mL) were within normal limits. ECG and spirometry were unremarkable. Given the minute, peripheral nodules, bronchoscopic biopsy was considered technically infeasible. Video‐assisted thoracoscopic wedge resection of the right upper lobe was performed without complications; the patient was discharged on postoperative Day 3.

**FIGURE 2:**
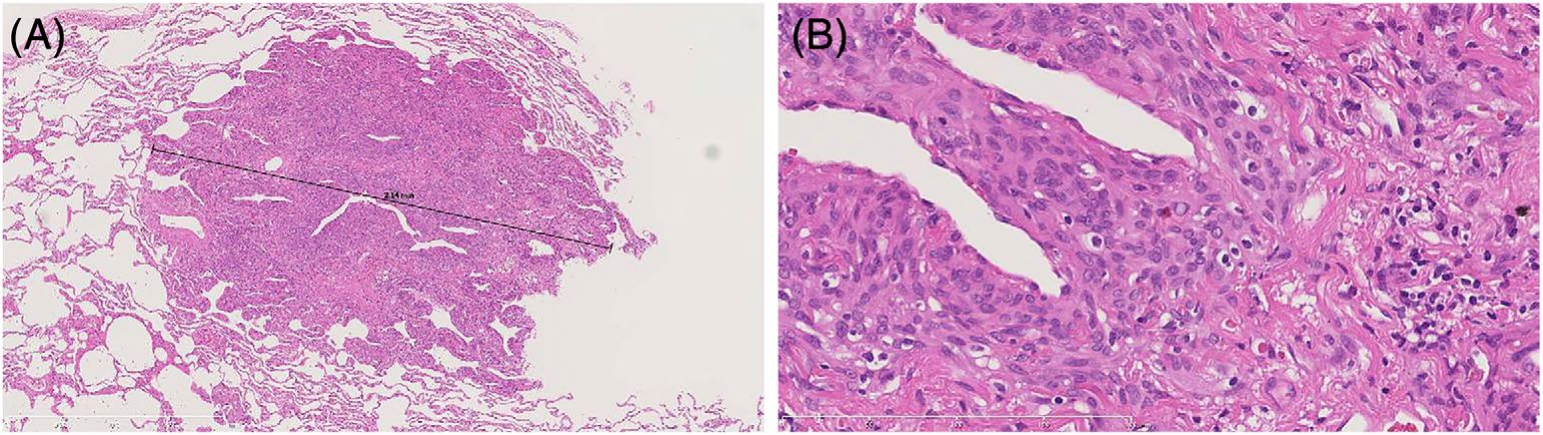
Lung wedge resection. (A) Low‐power H&E showing a well‐circumscribed subpleural nodule. (B) High‐power H&E demonstrating spindle‐to‐ovoid cells in whorled arrangements without atypia, mitoses, or necrosis.

**FIGURE 3:**
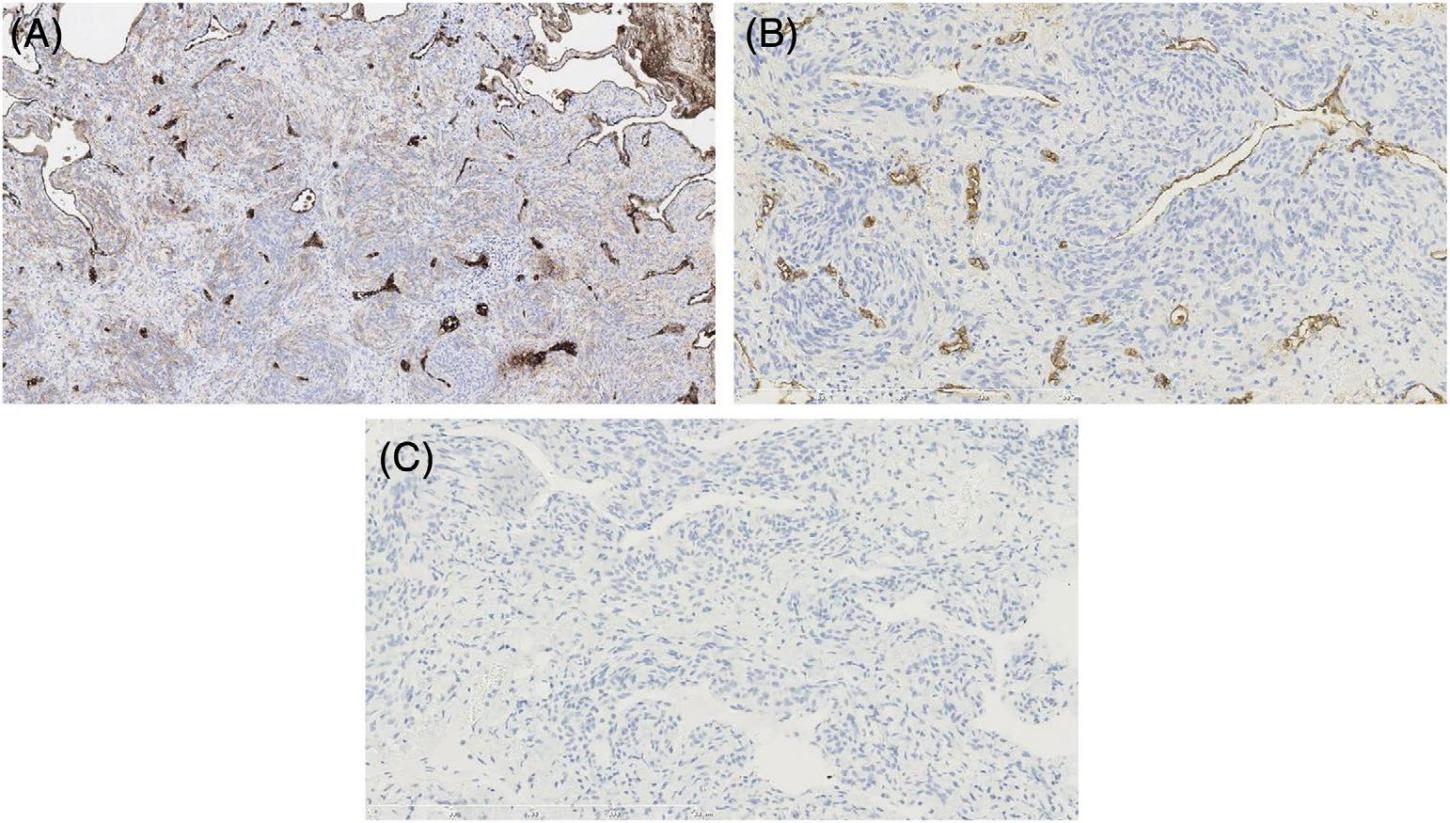
Immunohistochemistry. (A) Vimentin: Diffuse cytoplasmic positivity in tumor cells. (B) EMA: Negative in tumor cells (internal controls present). (C) Chromogranin A: Negative (scale bars as indicated).

Grossly, multiple small, firm subpleural nodules were present. Microscopically, nodules comprised spindle‐to‐ovoid cells arranged in whorled patterns with inconspicuous nucleoli and absent mitotic figures or necrosis (Figure 2A,B). Immunohistochemistry showed vimentin positivity and negativity for EMA, cytokeratin (AE1/AE3), SMA, and chromogranin (Figure 3). Postoperative brain MRI detected no intracranial lesion. The patient remains asymptomatic with no new lesions or growth over 3‐year surveillance CTs at regular intervals.

## 3 Discussion

This case exemplifies DPM presenting as diffuse pulmonary micronodules—a pattern easily mistaken for metastatic spread or granulomatous disease [1, 2]. Key differentials and our rationale for exclusion include: (i) miliary tuberculosis—lack of constitutional symptoms/lymphadenopathy and long‐term stability; (ii) sarcoidosis—absence of perilymphatic distribution/mediastinal adenopathy; (iii) hematogenous metastases—normal tumor markers, no primary tumor, and durable radiologic stability; and (iv) lymphoproliferative disorders—no systemic features or interstitial changes.

Immunohistochemistry supported meningothelial‐like differentiation: vimentin positive with epithelial/neuroendocrine markers negative. While EMA is typically positive in DPM/MPMN and PPM, occasional weak/negative staining is reported; technical variables (antibody clone/epitope retrieval) and low antigen expression may contribute [5, 6]. Where tissue is available, PR and SSTR2A staining can further substantiate the diagnosis and align with the literature [5, 6].

Although molecular testing is not mandatory for diagnosis, recurrent alterations in NF2, TRAF7, KLF4, AKT1, and SMO characterize meningiomas and may aid in ambiguous cases or provide prognostic context [7]. We acknowledge the absence of molecular testing as a limitation.

## 4 Conclusion

DPM should be considered in the differential of diffuse pulmonary micronodules, particularly in asymptomatic, stable cases. Surgical biopsy remains pivotal when bronchoscopic sampling is impracticable [1, 2].

## Author Contributions

Conceptualization: E.K. Investigation (surgery/clinical): E.K. Radiology (image acquisition/interpretation): H.O. Pathology and immunohistochemistry: T.N. Validation: Y.S. Data curation: E.K. Writing – original draft: E.K. Writing – review and editing: Y.S. Supervision: Y.S. Guarantor: E.K.

## Conflicts of Interest

The authors declare no conflicts of interest.

## Data Availability

The data that support the findings of this case (clinical imaging and histopathology files) are not publicly available to protect patient privacy. De‐identified data may be available from the corresponding author on reasonable request and with approval from the authors' institution.
